# The Side Effects and Adverse Clinical Cases Reported after COVID-19 Immunization

**DOI:** 10.3390/vaccines10040488

**Published:** 2022-03-22

**Authors:** Roshina Rabail, Waqar Ahmed, Madiha Ilyas, Muhammad Shahid Riaz Rajoka, Abdo Hassoun, Abdur Rauf Khalid, Moazzam Rafiq Khan, Rana Muhammad Aadil

**Affiliations:** 1 National Institute of Food Science and Technology, University of Agriculture, Faisalabad 38000, Pakistan; roshina.rabail@gmail.com (R.R.); 729waqar@gmail.com (W.A.); mrkhan_ft@yahoo.com (M.R.K.); 2 Department of Nutritional Sciences, Government College Women University, Madina Town, Faisalabad 38000, Pakistan; madihailyasrana@hotmail.com; 3 Food and Feed Immunology Group, Laboratory of Animal Food Function, Graduate School of Agricultural Science, Tohoku University, Sendai 980-8572, Japan; shahidrajoka@yahoo.com; 4 Sustainable AgriFoodtech Innovation & Research (SAFIR), 62000 Arras, France; a.hassoun@saf-ir.com; 5 Syrian Academic Expertise (SAE), Gaziantep 27200, Turkey; 6 Department of Livestock and Poultry Production, Faculty of Veterinary Sciences, Bahauddin Zakariya University, Multan 60800, Pakistan; dr.abdurrauf@hotmail.com

**Keywords:** COVID-19 vaccination side effects, AstraZeneca side effects, Pfizer side effects, Moderna side effects, Sinopharm side effects, Sputnik V side effects

## Abstract

COVID-19 remains a deadly disease that poses a serious threat to humanity. COVID-19 vaccines protect the public and limit viral spread. However, public acceptance is significantly dependent on the efficacy and side effects (SEs) of the vaccinations being produced. Four important mechanisms have been examined for COVID-19 vaccines: DNA-based, mRNA-based, protein-based, and inactivated viruses. Vaccination safety research was formerly limited to manufacturer-sponsored studies, but numerous additional cross-sectional survey-based studies conducted globally have contributed to the generation of vaccine-related safety data reports. Twenty-seven studies and twenty-four case reports published-up till 2021 were overviewed for the presentation of SEs and their severity. Injection site pain remained the most dominant localized SE, while headache and fatigue were the most prevalent systemic SEs. Most studies reported that all vaccinations were safe, with very little or no adverse effects, but the nature of SEs was reported to be more persistent in DNA- and mRNA-based vaccines, while inactivated viral vaccines were associated with longer-duration SEs. Overall, SEs were found to be more dominant in women and youngsters. Case reports of adverse reactions have also been documented, but there is still a need to find out their pathological linkage with the COVID-19 vaccination.

## 1. Need for Vaccination against COVID-19

Vaccines can be used to control infectious diseases, and are well known to be effective in fostering a long-lasting immune memory. Vaccines currently save the lives of 2–3 million people each year. When recommending or rejecting vaccinations, the adverse effects on people’s trust in healthcare professionals should be considered [1]. Severe acute respiratory syndrome-coronavirus-2 (SARS-CoV-2) triggered a pandemic. The first cases were reported in Asia, which were then spread to other parts of the globe [2]. The COVID-19 infection is caused by SARS-CoV-2, against which immunity can be obtained either by natural or preventative immunization of the population at the mass level [3]. The COVID-19 pandemic has introduced a challenge never before seen in the history of humankind for a secure and fearless existence [4], as it has directly afflicted millions of people around the globe. International agencies have recently discovered that poverty and hunger may kill far more people than COVID-19 victims [5]. However, COVID-19 remains a lethal disease that poses a significant threat to humanity. Many significant pharmaceutical and non-pharmaceutical companies have spent significant effort and money in the fight against this disease in recent years [6], as there was an immediate need to find a long-term therapeutic strategy, such as immunization [2]. Since the start of the coronavirus pandemic, experts have been working diligently to discover a way to stop the virus from spreading. The most pressing objective in the aftermath of such a public health crisis was the development of an effective vaccination [7], as vaccinating the global population is the most effective strategy to combat the COVID-19 pandemic. The promotion of these preventative methods, such as vaccination, is ethical and cost-effective and should be actively pursued by all physicians, particularly in the case of immune-compromised patients, e.g., patients with systemic sclerosis, who should be provided with the means to better protect their quality of life [8]. However, the public’s apprehension regarding the available vaccines is a significant obstacle in the fight against the spread of COVID-19 [9]. 

Vaccines against COVID-19 protect the general public and limit viral spread [2]. The four key mechanisms investigated in this paper for the COVID-19 vaccines are inactivated virus, DNA, mRNA, and protein-based vaccines. DNA-based vaccinations transfer the DNA coding for the SARS-CoV-2 spike protein using viral vectors into cells; mRNA vaccines typically deliver mRNA into the cells via a lipid nanoparticle; protein vaccines are based on the spike proteins or their particles, and certain other vaccinations are based on inactivated viruses [10]. Vaccines are one of the most effective therapies for combating COVID-19, saving millions of lives each year. However, the ideal option is a vaccine that is efficacious, safe and does not cause serious adverse responses. Due to the lack of viable and licensed COVID-19 treatments, a vaccination development race has started, with 259 COVID-19 vaccine projects beginning on 11 November 2020. The fast development of vaccines has increased the possibility of vaccine-related safety concerns [11]. Many countries are developing vaccines in the aftermath of the COVID-19 pandemic to protect their populations [12]. Many vaccines have been made available earlier than planned due to their pressing need. New technology has also aided the development of far more effective vaccines; however, their potential adverse effects must be considered [6]. 

## 2. Side Effects (SEs) and COVID-19 Vaccination Resistance

The development of a COVID-19 vaccine was viewed as a critical strategy for ending the pandemic. Public acceptance, on the other hand, is based on people’s ideas and perceptions of the vaccination [13]. The rates of vaccine hesitation and rejection are still higher, which may require legislative changes to make the vaccination program profitable [12]. One possible cause behind neglecting immunization could be the negative emotions such as anxiety, depression, anger, and irritability which have already been observed in many studies during quarantine periods. This occurs because psychosocial disturbances, such as relational loss and social rejection, cause changes in mind–body interplay [14]. Such hesitation has been linked to more negative beliefs that the vaccination would cause SEs or be unsafe [15]. The COVID-19 vaccine’s adverse effects play a critical role in public trust in the vaccination and its administration technique [3]. An online self-administered questionnaire, completed in May 2020 among the Saudi population regarding vaccination views and the potential barriers preventing people from becoming vaccinated against COVID-19, cited SEs as major obstacles to vaccine uptake [13]. Similarly, in another study, seven attributes were evaluated to create vaccine choice sets: vaccine effectiveness, SEs, accessibility, number of doses, vaccination sites, length of vaccine protection, and a fraction of acquaintances vaccinated. Although all seven factors were found to have a substantial impact on respondents’ vaccination decisions, and vaccination SEs were among the most relevant factors [16,17], the likelihood of a serious adverse SE was found to be a modest but significant cause of vaccination rejection. In comparison to rates of 1/1,000,000 general SEs, a significant adverse SE rate of 1/100,000 was more likely to discourage vaccine usage [18]. COVID-19 vaccinations have been expedited through the review process due to the lack of safety data. COVID-19 vaccines had a low level of public acceptance of 37.4%. Therefore public health officials must implement systematic interventions to reduce vaccine hesitancy and improve vaccine acceptance [19] and more studies are required to identify the benefits and SEs.

## 3. Selection and Collection of Data

The latest current available literature on COVID-19 immunization, COVID-19 vaccination updates, the studies on their reported SEs, updates on case reports presented with serious adverse SEs, their approval, authorization, and clinical phases were explored on Google scholar, Scopus, and Science Direct using the keywords “COVID-19 vaccination” or “COVID_19 immunization” or “SARS-CoV-2 vaccination” or “SARS-CoV-2 immunization” and “Side effects” or “Adverse effects” or “Safety studies”, available up to November 2021.

## 4. COVID-19 Vaccinations’ Approval/Authorization

Scientists across the world are working faster than ever to design and create vaccinations that can prevent the spread of COVID-19, with 21 vaccines already being distributed in countries around the world [20]. COVID-19 vaccines were previously available in three forms: messenger RNA-based vaccinations, adenoviral vector vaccines, and inactivated whole-virus vaccines. Some of them have passed phase 3 safety and efficacy trials and are now commonly utilized for COVID-19 infection prophylaxis [21], but now protein subunit forms of COVID-19 vaccines are also under consideration [22,23]. Thus far, the US Food and Drug Administration (FDA) has granted emergency use approval for three new COVID-19 vaccines: two messenger RNA-based vaccines (Pfizer and Moderna), and one adenoviral vector vaccine (Janssen) has received FDA approval [24]. In the event of a public health emergency, emergency use authorization (EUA) is provided for unlicensed medications and vaccines. The FDA Director General is authorized to issue the EUA under Executive Order No. 121; the following vaccinations have received this license: Pfizer, AstraZeneca, Coronavac/Sinovac, Sputnik V, Janssen, Covaxin, Moderna, and Sinopharm [25]. Seven vaccinations are listed on the WHO emergency use listing (EUL). These include Moderna, Pfizer, Janssen, AstraZeneca, Covishield, Sinopharm, and Sinovac [26]. Two vaccines (Moderna and Janssen) have launched successful phase 4 clinical trials. The United States authorizes Moderna for use on anyone over the age of 18, while the European Medicines Agency has given approval for youngsters aged 12 to 17 in July 2021. Four vaccinations (AstraZeneca (Cambridge, United Kingdom), Pfizer (New York, United States), Sinovac (Beijing, China), and Cansino (Tianjin, China) have advanced to phase 3 as part of the WHO’s vaccine solidarity study. There are now 128 COVID-19 vaccine candidates in clinical trials and 194 candidates in pre-clinical research across the world [20]. Eleven vaccines based on their authorization, approval, efficacy level, and utilization have been listed in Table 1. 

## 5. Studies Reporting SEs of COVID-19 Immunizations

**Figure 1:**
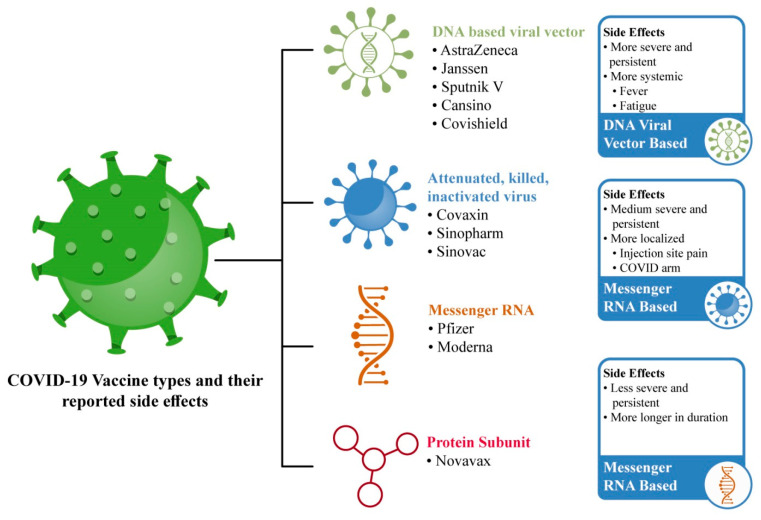
Most-utilized COVID-19 vaccinations and their frequently reported SEs.

Previously, vaccination safety research only came from manufacturer-sponsored studies, but many other cross-sectional survey-based studies around the world have helped in the generation of vaccine-related safety data reports [3]. COVID-19 vaccine types and their SEs are illustrated in Figure 1.

### 5.1. Messenger RNA Based COVID-19 Immunization

The FDA granted Emergency Use Authorization (EUA) to two two-dose mRNA vaccines: BNT162b2 from Pfizer–BioNTech, for people aged ≥16 years; and mRNA-1273 Moderna for people aged ≥18 years [32]. Both vaccines employ either lipid nanoparticle delivery technology or a modified mRNA-delivery mechanism. Modified mRNA is used to encode the COVID-19 spike proteins, with mutant mRNA being added to lock them into the three-dimensional structure required to cause an interaction between the spike proteins and virus-neutralizing antibodies. They have a higher safety profile than other viral vaccines since they are not created with actual infections and are not incorporated into host DNA [28]. Both have been considered safe during pregnancy [29]. Messenger RNA vaccinations generate milder, less-frequent systemic adverse effects, but more localized SEs [9,33]. 

#### 5.1.1. Pfizer–BioNTech (BNT162b2) COVID-19 Vaccination

**Figure 2:**
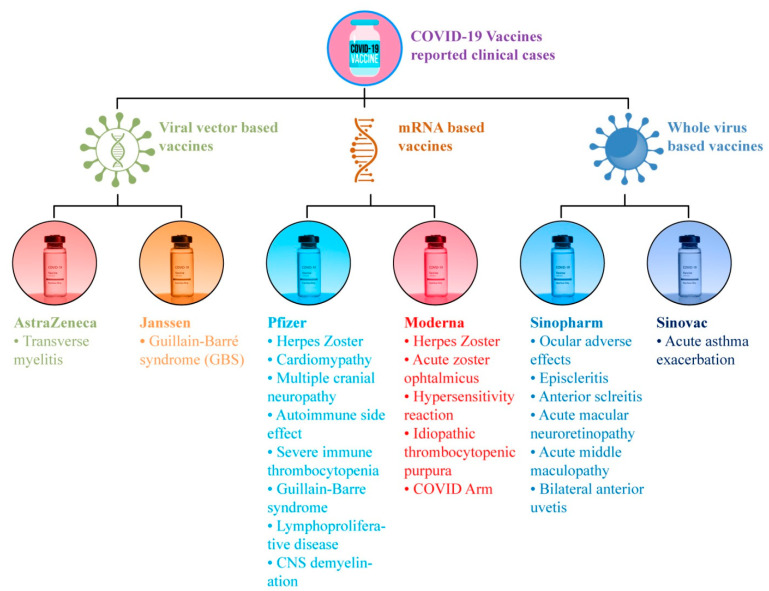
Clinical cases for adverse effects after COVID-19 vaccinations.

Millions of people worldwide have been immunized with the Pfizer–BioNTech vaccination [24]. The Pfizer–BioNTech (BNT162b2) vaccine has shown good safety and efficacy in phase 3 trials and reduces the chances of SARS-CoV-2 infection after approximately 12 days of vaccination [34]. The Pfizer–BioNTech vaccine was associated with considerably greater rates of all forms of adverse reactions [35]. A selection of the minor SEs, which have been highlighted in ten studies on the Pfizer–BioNTech vaccine, is listed in Table 2. Among these eleven studies on Pfizer, eight studies reported headache; seven studies reported weakness/fatigue and myalgia/muscle/body pain; six studies reported local injection site/shoulder pain, chills/feeling cold, and fever; four studies reported enlarged lymph nodes and joint pain/arthralgia; three studies reported nausea/vomiting/GIT disturbances; one study reported cutaneous urticarial/morbilliform eruptions, weakness, hand numbness, mucosal lesions, taste disturbances, skin burning, rash, allergic reactions, dry cough, sore throat, brain fog, and decreased sleep quality. On the other hand, the prevalence of major or complex SEs has also been reported in these nine studies, including one study which reported adverse skin reactions, i.e., chilblains; zoster, herpes simplex, pityriasis rosea, etc.; two studies, which reported severe allergic responses such as anaphylaxis; one study which reported thromboembolic events such as a cerebrovascular accident, myocardial infarction, pulmonary embolism, acute hypertension (over 210/105mm Hg), and Bell’s palsy. While exploring the data, available in the form of clinical case reports in Table 3, a total of twenty cases reported clinical complications which may have been linked with the Pfizer vaccination. Among these cases, eight belong to the onset of acute zoster ophthalmicus/varicella-zoster virus reactivation/shingles (herpes zoster); three belong to the lymphoproliferative disease and autoimmune adverse reactions (antineutrophil cytoplasmic autoantibody and severe immune thrombocytopenia); two cases pertain to acute immune thrombocytopenia; and one involves a clinical complication for takotsubo cardiomyopathy, multiple cranial neuropathies, and Guillain-Barré Syndrome. Some of the reported clinical cases for the COVID-19 vaccine and their SEs are illustrated in Figure 2.

#### 5.1.2. Moderna (mRNA-1273) COVID-19 Vaccination

The Moderna vaccine, similar to the Pfizer vaccine, received FDA approval in its early clinical efficacy trials [29,63]. The SEs recorded for this vaccination are reasonably similar to those reported for other vaccines [63]. The minor SEs of Moderna has been highlighted in the five studies listed in Table 2. The most reported SEs include injection site pain, headache, fatigue, muscle pain, malaise, chills, joint pain, mucosal lesions, oral paresthesia, taste disturbance, pruritus, rash, itchy sensations in the mouth and throat, sensations of throat closure, muscles spasms, anorexia, decreased sleep quality diarrhea, flushing, nasal stiffness, and respiratory symptoms. Local injection site reactions, such as urticarial eruptions and morbilliform eruptions, have also been reported. While exploring the data available in the form of clinical case reports in Table 3, a total of fifteen cases have been reported thus far, which report complaints of clinical complications associated with the Moderna vaccination. Among these, there are two cases of severe hypersensitivity reactions with generalized pruritus, urticaria, tachycardia, chest, and neck urticarial, and mild facial angioedema; two complications of herpes zoster ophthalmicus (HZO) reactivation; one case of idiopathic thrombocytopenic purpura; four cases of COVID arm with erythematous plaques; three acute kidney complications with glomerulonephritis and proteinuria; two cases of encephalopathy; and one case of acute immune thrombocytopenia with generalized petechiae. 

### 5.2. Viral Vector-Based COVID-19 Immunization

The non-replicating adenoviruses used in viral vector-based vaccines are safe for humans. This vaccine’s technique has been in use for decades. Two distinct adenoviruses (AD16 and AD5) are employed, one in each dose of vaccine, with a 21-day interval [76]. The prevalence of systemic SE was higher in the AZD-1222 vaccine than in the Sputnik V and Covaxin vaccines [44]. 

#### 5.2.1. AstraZeneca (ChAdOx1 nCoV-19) COVID-19 Vaccination

The AstraZeneca (ChAdOx1 nCoV-19) vaccine has shown good safety and efficacy in phase 3 trials and reduces the chances of SARS-CoV-2 infection after about 12 days of vaccination [34]. The AstraZeneca ChAdOx1 nCoV-19 (AZD1222) vaccine has been linked to thrombosis with thrombocytopenia syndrome (TTS) in 3/100,000 people, with high fatality rates reported in many countries. In Australia, the potential risks of the AZD1222 vaccine in younger adults who are unlikely to die from COVID-19 may outweigh the benefits [77]. A selection of the minor SEs that have been highlighted in five studies on the AstraZeneca vaccine is listed in Table 2. All five studies reported headache and myalgia/muscle/body pain; four studies reported weakness/fatigue, chills, nausea, diarrhea; three studies reported local injection site/shoulder pain and fever; two studies reported joint pain and tachycardia; and one study reported dry cough, shortness of breath, nasal discharge, swollen armpit, bruising, rash, allergic reactions, red felts on face, skin, oral mucosal lesions, and taste disturbances. The severe SEs reported in these studies were severe hypotension, generalized body aches, shortness of breath, and fever with a temperature greater than 39°C. One study presented two clinical cases of transverse myelitis, a neurological disorder that might have been linked to the AstraZeneca vaccination. 

#### 5.2.2. Covishield (AZD1222)

Covishield is a COVID-19 vaccine developed in India. Covishield is currently in use and has nearly 90% effectiveness. It was developed by Oxford–AstraZeneca and is manufactured by the Serum Institute of India (SII) in Pune, Maharashtra. It employs the same technology that was used to develop vaccines for viruses such as Ebola, a chimp adenovirus, namely, ChAdOx1. This technology has been modified to carry the COVID-19 spike protein into human cells. A web-based survey study reported the SEs of the Covishield vaccine. The prominent SEs were mild fever (28.91%), myalgia (26.43%), cold and cough (8.16%), headache (6.74%), and local injection site pain (3.37%). Less-prevalent SEs included fatigue, diarrhea, rigors, joint pain, and nausea. Only 0.70% of recipients claimed severe symptoms with admission and observation in clinical setups [53].

#### 5.2.3. Janssen (Ad26.COV2.S) COVID-19 Vaccination

The Janssen vaccine is based on the genetic modification of inactivated adenoviruses by the deletion of the E1 gene, which is replaced with the spike gene [78]. A VAERS-based study reported 64 anxiety-related events, as elaborated in Table 2. Other SEs included tachycardia, hyperventilation, dyspnea, chest pain, paresthesia, light-headedness, hypotension, headache, pallor or syncope, and fainting. Three clinical cases of severe immune thrombocytopenia (ITP), severe cutaneous adverse reaction, and Guillain-Barré syndrome (GBS) have been reported in its major complications, as shown in Table 3. 

#### 5.2.4. Sputnik V (Gam-COVID-Vac) COVID-19 Vaccination

Russia’s first authorized vaccination was developed and manufactured wholly in the country, and its name alludes to the 1950s space race. To create the vaccine, the adenoviruses are mixed with the SARS-CoV-2 spike protein, which causes the body to respond with an immunological response [79]. Sputnik V was registered as Gam-COVID-Vac by the Russian Ministry of Health in August 2020, and it has been delivered in 61 countries globally since December 2020 [48]. A total of five studies have been added here; the SEs of Sputnik V are reported in Table 2. All five studies reported injection site pain; three studies reported fatigue, headache, swelling, and chills; four studies reported muscle or body pain and fever; two studies reported joint pain nodules or lymph nodes, diarrhea, and redness; and one study reported drowsiness, warmth, asthenia, malaise, insomnia, nausea, vomiting, and pruritus. 

### 5.3. Inactivated COVID-19 Immunization

#### 5.3.1. Covaxin Vaccine (BBV152)

India has produced a COVID-19 vaccine—namely, COVAXIN—that is an inactivated vaccine produced by Whole-Virion Inactivated Vero Cell-derived platform technology. It does not require reconstitution or sub-zero storage and comes in ready-to-use multi-dose vials that are stable between 2 and 8 °C. It cleared Phase I and II human clinical trials in July 2020. Vaccine-induced antibodies, according to the National Institute of Virology, can neutralize the UK variant strains as well as other heterologous strains. The effectiveness of the Covaxin vaccine is nearly 81% [53]. Local injection site pain, fatigue, muscular pain, and fever were described in a single investigation on the SEs of a Covaxin dose [44]. 

#### 5.3.2. Sinovac

The most common local SE 54.6% was pain, while the most common systemic SEs were fatigue 39.2% and headache 34.1%. Two-thirds of individuals who were vaccinated reported at least one local or systemic SE, with women and people under the age of 35 being the most affected. Individuals who worked more than 8 h a day felt the vaccine’s local adverse effects, such as increased hunger and weariness, more acutely [54]. A clinical case for severe acute asthma exacerbation was reported as a possible adverse effect of the Sinovac COVID-19 vaccination (Table 3).

#### 5.3.3. Sinopharm (BBIBP-CorV/Vero Cells) COVID-19 Vaccination

The Sinopharm vaccine is a complete viral inactivated vaccine manufactured from Vero cells. These cells replicate the SARS-CoV-2 virus, which is subsequently treated with beta-propiolactone, which deactivates the virus by binding to its genes [28]. Sinopharm post-vaccination SEs remain modest and predictable for the first and second doses, with no cases of hospitalization, which help to reduce vaccine hesitancy [51,80]. Sinopharm vaccine recipients had a longer duration of adverse effects. The majority of these adverse effects are minor and curable [35]. A total of six studies have been documented here for SEs of Sinopharm in Table 2. All six studies reported headache; five studies reported fatigue; three studies reported injection site pain, body, or muscle pain; five studies reported headache; four studies reported fever; two studies reported myalgia, cough, flu, or nasal discharge, shortness of breath, abdominal pain, and diarrhea; one study reported lightheadedness, weariness, multiple bruises with productive bleeding; swelling in the legs and arms; chest pain, chills, arm pain, tenderness, lethargy, shivering, and rigors; malaise, myalgia, arthralgia, joint pain, sore throat, nausea, anxiety, and dizziness. Seven clinical cases of ocular adverse effects (episcleritis, anterior scleritis, acute macular neuroretinopathy, acute middle maculopathy, subretinal fluid); one case for anterior uveitis associated with juvenile idiopathic arthritis (JIA); and one case for bilateral anterior uveitis with reduced visual acuity in both eyes has been documented in the literature, as shown in Table 3.

## 6. COVID-19 Vaccinations’ Reported Minor Side Effects

As elaborated in Table 1, the most-prevalent minor SE reported after COVID-19 vaccinations were localized reactions in the form of local injection site reactions, injection site/shoulder pain swelling, and soreness surrounding the area, whereas the most-prevalent generalized SEs were headache, fever, sweating, chills, tiredness, and fatigue. There were minor SEs in the nose, throat, and oral cavity, including throat infection or irritations, breath tightness, nasal stuffiness, flu-like symptoms, sensations of throat closure, oral mucosal lesions, paresthesia, and taste disturbance. The minor SEs of musculoskeletal symptoms included joint pain, muscular spasm, whole-body aches/myalgia, osteoarticular pain, back pain, and neck pain. The SEs of skin included skin rashes or allergic responses of urticarial eruptions, morbilliform eruptions, and pruritus. The minor SEs of the gastrointestinal system included nausea, vomiting, and diarrhea, while other SEs reported include fast heartbeat, dizziness, flushing, palpitations, brain fogging, mental confusion, anorexia, decreased sleep quality, drowsiness, hand numbness, enlarged lymph nodes, etc.

## 7. COVID-19 Vaccinations’ Reported Major Side Effects

The adverse or major-severity SEs in Table 1 and Table 2 reported from COVID-19 vaccinations on skin included zoster or herpes simplex flares such as varicella-zoster virus reactivation, chilblains, cosmetic filler reactions, and pityriasis rosea. The major SEs of the cardiovascular system (CVS) reported in studies include thromboembolic events such as thrombosis, cerebrovascular accidents, myocardial infarction, takotsubo cardiomyopathy, and pulmonary embolism. The major SEs of the central nervous system (CNS) included CNS demyelination, multiple sclerosis, syncope, transverse myelitis, encephalopathy, stroke, and acute disseminated encephalomyelitis. The major SEs of musculoskeletal system included Guillain-Barré syndrome, Bell’s palsy and facial palsy. Ocular adverse effects included episcleritis, anterior scleritis, acute macular neuroretinopathy, acute middle maculopathy, subretinal fluid, and anterior uveitis associated with juvenile idiopathic arthritis (JIA). Severe immune system disturbances and autoimmune side effects were also observed, such as antineutrophil cytoplasmic autoantibody (ANCA)-associated vasculitis of acute kidney injury, severe immune thrombocytopenia, lympho-proliferative disease, hypersensitivity reaction, and severe cutaneous adverse reaction with panhypopituitarism secondary to craniopharyngioma resection. Furthermore, among adverse reactions, COVID arm with pruritic, erythematous plaques were reported. However, the link between the mildness and severity of SEs, along with the mechanism involved and other risk factor associations, is still under debate and requires proper investigative studies.

## 8. Conclusions

Twenty-seven studies on SEs and twenty-four case reports reporting fifty-six various clinical adverse effects of various COVID-19 vaccines have been overviewed in this study. Conclusive outcomes for local and systemic SEs revealed that the mRNA-based vaccines were more likely to be linked with localized adverse effects (e.g., injection site discomfort), while viral vector-based vaccines were found to be more common in imparting systemic SEs (e.g., headache/fatigue). Individuals who received Pfizer and AstraZeneca vaccines exhibited higher overall local site reactions when compared to those who received the Sinopharm vaccine. Regarding the SE severity, persistency, and duration, the AstraZeneca vaccine was found to have more persistent and severe SEs compared to other vaccines such as Pfizer; additionally, Pfizer’s SEs were dominant in comparison to the Sinopharm vaccine. Overall, the vaccinations from Pfizer, AstraZeneca, and Sinopharm were judged to be safe, with Sinopharm having a lower number of adverse effects than the other vaccines. Pfizer was associated with greater rates, while Sinopharm was associated with longer-duration SEs. On the other hand, the outcomes for age and gender revealed that females and youngsters, particularly individuals ≤ 43 years old, were found to be linked with a higher risk of adverse effects by both mRNA-based and viral vector-based immunizations. Sputnik V and Covaxin reported fewer SEs and better tolerance levels in the elderly. Injection site pain, fatigue, headache, muscle or body pains, and fever were the most reported SEs. Certain adverse effects, such as cutaneous reactions, herpes reactivation, ocular adverse effects, Bell’s palsy, lymph nodes, anaphylaxis, thrombosis, myocardial infarction, cardiomyopathy, severe hypotension, multiple sclerosis relapse, syncope, stroke, GBS, facial palsy, myelitis, autoimmune SEs, acute disseminated encephalomyelitis, and multiple cranial neuropathies, were also reported in these studies. Overall, studies found that all immunizations were safe, with very few or no SEs; however, the form of SEs was shown to be more persistent in DNA- and mRNA-based vaccines, whereas inactivated viral vaccines were associated with longer-duration SEs. Overall, SEs were shown to be more prevalent in women and youngsters. Certain instances of adverse responses have also been observed, although their pathological relationship with COVID-19 immunization has yet to be determined.

## Figures and Tables

**Table 1:** Most-utilized vaccinations against COVID-19.

Local/Given Name	Codename	Type	Origin: Company/Country	Approval/Authorization	Efficacy Trials Phase II/III/IV	References
Pfizer	BNT162b2 Vaccine	Uridine nucleoside modified mRNA (encodes the receptor-binding domain or full-length version of SARS-CoV-2 spike protein)	BioNTech (Germany) in collaboration with Fosun Pharmaceuticals (Shanghai, China) and Pfizer (Canada)	FDA-approved; FDA Emergency Use Authorization (EUA); WHO-approved in 103 countries; among first vaccines to get EUA in December 2020	95% efficacy. Safe during pregnancy. 38 trials in 20 countries. Phase IV registered on 27 September 2021	[6,20,25,26,27,28,29,30]
Moderna	mRNA-1273 Vaccine	mRNA-based vaccine designed to express the coronavirus spike protein	Massachusetts (the United States) along with the National Institute of Allergy and Infectious Diseases and Biomedical Advanced Research and Development Authority	FDA-approved; FDA (EUA); WHO-approved in 76 countries; among first vaccines to get EUA in December 2020	94% efficacy; safe during pregnancy. 31 trials in 7 countries; phase II in August 2021; phase IV launched in June 2021	[6,20,25,26,27,28,29,30]
Janssen	Ad26.COV2.S	Adenoviral-based vaccine	Johnson and Johnson/the United States, the Netherlands.	FDA-approved; FDA (EUA); WHO-approved in 75 countries	13 trials in 3 countries. Phase IV launched in June 2021	[6,20,25,26,28]
AstraZeneca/Vaxzevria	AZD1222 Vaccine/ChAdOx1	Chimpanzee Adenovirus-Vectored vaccine	Oxford University under British pharmaceutical company	FDA (EUA); WHO emergency use listing (EUL) WHO-approved in 124 countries	70% efficacy. 46 trials in 3 countries. Phase II/III was completed in June 2021; Phase IV was registered on 27 September 2021 [26]	[4,16,21,22,23,24,26]
Sputnik V	Gam-COVID-Vac	Adenoviral-based vaccine	Gamaleya National Research Centre of Epidemiology and Microbiology (Moscow, Russia)	FDA (EUA)	91.6% efficacy;	[25,27,28,30]
Convidecia/CanSino	Ad5-nCoV Vaccine	The recombinant vaccine which involves replication-defective adenovirus type 5 as vector	Tianjin, China in collaboration with the Beijing Institute of Biotechnology in the Academy of Military Medical Sciences	-	90% efficacy. Phase IV registered in May 2021	[20,27,30]
Covaxin Vaccine	BBV152	Inactivated vaccine candidate; deactivated rabies vaccine as a vehicle for coronavirus proteins	Bharat Biotech (India) in collaboration with Thomas Jefferson University of Philadelphia, Indian Council of Medical Research (ICMR) and National Institute of Virology (Pune, India)	FDA (EUA)	-	[25,27]
Sinopharm	New Crown COVID-19 Vaccine	Inactivated vaccine candidate	Wuhan Institute of Biological Products, (China)	FDA (EUA); WHO (EUL); WHO-approved in 68 countries	79.34% efficacy. 15 trials in 9 countries	[20,25,26,27,28,30]
Sinovac/CoronaVac	CoronaVac Vaccine	Adsorbed (inactivated) vaccine	Sinovac Life Sciences Co. Ltd. (China) in collaboration with Instituto Butantan	WHO emergency use listing (EUL); WHO-approved in 41 countries	65% efficacy; 22 trials in 2 countries. Phase IV registered on 27 September 2021	[20,26,27,30]
Covishield	AZD1222	(Oxford/ AstraZeneca formulation)	Serum Institute of India	WHO-approved in 46 countries?	2 trials in 1 country	[26]
Novavax	NVX-CoV2373	Antigenic components (spike (S) protein) generated in vitro (Viral subunit)	-	-	-	[30,31]

**Table 2:** COVID-19 vaccinations and their reported SEs.

Vaccine Name	Methodology	Participant/Area	Date/Duration	Minor Side Effects	Major Side Effects	Duration	Concluding Remarks	References
Pfizer– BioNTech COVID-19 (17%) and Moderna (83%)	A collaborative study between the American Academy of Dermatology and the International League of Dermatological Societies	414 cutaneous reaction cases	December 2020–February 2021	Local injection site reactions, urticarial eruptions, morbilliform eruptions.	Pernio/chilblains, cosmetic filler reactions, zoster, herpes simplex flares, pityriasis rosea.	-	The presence of a cutaneous reaction to the first vaccine dose, when it appears 4 h after injection, is not a contraindication to receiving the second dose of the Pfizer or Moderna vaccine.	[36]
Pfizer–BioNTech COVID-19	Google Form-questionnaire (online survey)	455 individuals Saudi Arabia inhabitants	10–21 January 2021	Injection site pain, headaches, flu-like symptoms, fever, tiredness, fast heartbeat, whole-body aches, difficulty breathing, joint pain, chills, drowsiness.	Bell’s palsy, lymph node swelling and tenderness.	-	-	[37]
Pfizer–BioNTech COVID-19	Pharmaceutical and Medical Devices Agency (PMDA) reported adverse events following immunization (AEFI)	578,835 doses	February–March 2021	-	181 suspected event reports of anaphylaxis and anaphylactoid symptoms (reporting rate: 8.1/100,000 doses)	-	In 171 of these 181 cases, women developed these symptoms.	[38]
Pfizer–BioNTech COVID-19 (64.5%) and Moderna (35.5%)	Cross-sectional trial with an independent online questionnaire	1245 HCWs	24 January–10 March 2021	Soreness, fatigue, myalgia, headache, chills, fever, joint pain, nausea, muscle spasm, sweating, dizziness, flushing, feelings of relief, brain fogging, anorexia, localized swelling, decreased sleep quality, itching, tingling, diarrhea, nasal stuffiness, palpitations.	-	-	-	[39]
Pfizer–BioNTech COVID-19 and Moderna	Vaccine Adverse Event Reporting System (VAERS); Food and Drug Administration Adverse Event Reporting System (FAERS)	Women aged ≤50 years with hormonal contraceptive use	19 March 2021	-	68 thromboembolic events (1 case per 222,951 vaccinated) thromboembolic events under investigation: thrombosis, cerebrovascular accident, myocardial infarction, pulmonary embolism.	1–6 days after vaccination	-	[40]
Pfizer–BioNTech COVID-19	Cross-sectional survey-based study	HCW in the Czech Republic	January–February 2021	Injection site pain, fatigue, headache, muscle pain, chills.	-	1 or 3 days	SEs more prevalent among the ≤43-year-old age group.	[7]
Moderna COVID-19 vaccine	CDC report (VAERS)	108 case reports of severe allergic reaction, including anaphylaxis	21 December 2020–10 January 2021	Pruritus, rash, itchy sensations in the mouth and throat, sensations of throat closure, and respiratory symptoms.	10 cases of anaphylaxis (2.5 anaphylaxis cases/million Moderna COVID-19 vaccine doses.	7–30 min after vaccination	-	[41]
AstraZeneca and Pfizer–BioNTech	Online survey	Total 705: Pfizer 196. AstraZeneca 509	-	Injection site pain, shoulder pain, muscle aches, headaches, fever, chills, weakness, nausea, vomiting, diarrhea in both vaccines. General stomach problems, osteoarticular pain, back pain, neck pain, drowsiness, feeling cold, fast heart rate and palpitations in AstraZeneca. Hand numbness, enlarged lymph nodes in Pfizer.	-	-	AstraZeneca causes more SEs. Pfizer had the same or more adverse reactions after the second dose; AstraZeneca’s second dose caused even stronger SEs.	[42]
Pfizer-BioNTech, Moderna and AstraZeneca	Online questionnaire	599 HCWs in the Federal Republic of Germany	February–April 2021	Injection site pain, headache, fatigue, muscle pain, malaise, chills, joint pain. More than one-sixth participants reported at least one oral side effect, including mucosal lesions, oral paresthesia, taste disturbance.	-	1–3 days	mRNA-based vaccines: more local SEs. Viral vector-based vaccine: higher systemic SEs. Females and the younger age groups are more associated with an increased risk of SEs.	[9]
Pfizer- BioNTech and AstraZeneca	COVID symptom study app	282103 individuals (aged 16–99 years)	8 December 2020–10 March 2021	Headache, fatigue, chills, shivering, diarrhea, fever, arthralgia, myalgia, nausea, pain, swelling, tenderness, itch, swollen armpit glands, redness, warmth, bruising, allergic reactions, rash, skin burning, red welts on face and lip.	-	1–2 days	Adverse effects are more frequently reported in younger individuals, women, and among those who previously had COVID-19.	[34]
Pfizer-BioNTech, AstraZeneca, and Sinopharm	An interactive web-based system interview with an electronic version of the questionnaire	1736 (18–86 years age). Pfizer 700. AstraZeneca 696; Sinopharm 340	1 January–10 April 2021	Fatigue, body pain, headache, muscle pain, fever, gastrointestinal effects (nausea, vomiting, anorexia, and diarrhea) in all three vaccines; tenderness or swollen lymph nodes in Pfizer; sweating, dizziness, dry cough, anxiety, shortness of breath, tachycardia, abdominal pain, sore throat, joint pain, nasal discharge in AstraZeneca	Six cases: (four- Pfizer and two AstraZeneca) admitted into the hospital due to severe hypotension, generalized body aches, shortness of breath, and fever of more than 39 °C. Four cases (2 swelling and severe allergic reaction of eyelids and 2 acute hypertension—over 210/105 mm Hg) in Pfizer vaccine.	1–2.5 days	Signs and symptoms are more persistent for AstraZeneca followed by Pfizer and less adverse with Sinopharm.	[43]
AstraZeneca, Sputnik-V and Covaxin		503 HCWs in Birjand (Iran)	21 February–7 March 2021	Injection site pain, fatigue, muscle pain, fever in all three vaccines.	-	-	SEs more persistent in female; Sputnik V and Covaxin reported lower SE occurrence in the elderly.	[44]
Sinopharm (51.1%) and Pfizer–BioNTech (48.9%)	Semi-structured interviews on a phone call	1004 Jordanian adults with no history of previous allergies	10 March–2 April 2021	Local and systemic effects: pain at the injection site, fatigue, headaches, myalgia, arthralgia, fever, rigors.	No serious cases of hospitalization.	-	Pfizer was associated with greater rates, while Sinopharm was associated with a longer duration of SEs.	[35]
Sinopharm	Google form	583 Iranian multiple sclerosis (MS) patients	1 May–22 May 2021	Malaise, fatigue, fever, shivering, body pain, headache.	Five recipients (0.9%) reported MS relapse.	-	-	[45]
AstraZeneca	Cross-sectional survey-based study	92 HCWs/Germany and the Czech Republic	March 2021	injection site discomfort, fatigue, muscle pain, chills, feeling unwell, nausea, headache	No serious cases of hospitalization/no blood disorder.	-	Chronic diseases were not associated with an increased risk of SE.	[7]
Janssen	VAERS	64 anxiety-related events	7–9 April 2021	tachycardia (rapid heart rate), hyperventilation (rapid breathing), dyspnea (difficulty breathing), chest pain, paresthesia (numbness or tingling), light-headedness, hypotension (low blood pressure), headache, pallor, or syncope	17 reports of syncope (fainting): 8.2 episodes per 100,000 doses.	Immediately after vaccination.	-	[46]
Sputnik V	Observational study	3236 reports out of 13,435 HCW	February–April 2021	pain in the injection site, fatigue, body pain, headache, fever, joint pain, chilling, drowsiness.	-	-	SEs more frequent in females and younger individuals.	[47]
Sputnik V	E-questionnaire	2558 people from San Marino; aged 18-89 years	4 March–8 April 2021	Local site pain, nodules, swelling, warmth, asthenia, headache, joint pain, muscle pain, chills, malaise, fever.	-	-	Sputnik V shows a strong tolerance profile in the population aged ≥60 years.	[48]
Sputnik V	An observational cohort study by the Ministry of Health of Buenos Aires City (CABA)	707 HCWs in Hospital Italiano de Buenos Aires	5–20 January 2021	Injection site pain, redness, swelling. Fever, diarrhea, muscle pain.	-	-	-	[49]
Sputnik V	LabelStudio data labeling tool to label the dataset	4579 entities	December 2020–April 2021	Injection site pain, fever, fatigue, headache, insomnia, nausea, vomiting, redness, pruritus, swelling, lymph nodes enlargement, diarrhea, chills.	-	-	-	[50]
Sinopharm	Cross-sectional survey	The United Arab Emirates	January–April 2021	Injection site pain, fatigue, headache, lethargy, fatigue, tenderness.	No serious cases of hospitalization.	-	SEs are more common in ≤49 years of age and females.	[11]
Sinopharm	Web-based cross-sectional survey.	≥18 years of age 2000 resident of Karachi	11 April–23 April 2021	Fever, muscle pain, chills, arm pain, breathlessness, diarrhea, cough, flu, fatigue, chest pain, headache, abdominal pain, swelling in the legs and arms, multiple bruises, productive bleeding.	-	-	-	[51]
Sinopharm COVID-19	Closed-ended questionnaire	155/400 Healthcare workers (HCWs) >18 years old; Khyber Teaching Hospital, Peshawar, Pakistan	March 2021	Pain at the injection site, weariness, headache, light-headedness, myalgia.	No serious cases of hospitalization.	-	More SE in the 24–42-year age group; Sinopharm vaccine has no or minor negative effects.	[52]
Covishield	Web-based self-report submission or vaccine event reporting system	5637 HCWs in India	16 January–6 February 2021	Mild fever, myalgia, cold, cough, headache, local pain, swelling, fatigue, diarrhea, rigors, joint pain, nausea	-	-	-	[53]
Sinovac	Questionnaire	355 nurses in Turkey	-	fatigue, headache, arthritis, sore throat, nausea, fever, vertigo, nasal flow, appetite changes, diarrhea, itchiness, abdominal pain, cough, changes in the mucosa, changes in taste sensation.	-	-	-	[54]
Different/Others	Online poll	2002	February 2021	Fever, dyspnea, flu-like illness, weariness, local reactions.	Few serious adverse effects such as anaphylaxis, in viral vector-based immunizations.	-	mRNA vaccines linked with higher but milder incidence of any side effect.	[33]
Different/Others	VAERS	9442 reports of adverse events in the United States	March 2021	Dizziness, headaches, discomfort, muscular spasms, myalgia, paresthesia.	Stroke (17), GBS (32), facial palsy (190), transverse myelitis (9 cases), acute disseminated encephalomyelitis (6 cases).	-	The rare occurrence of tinnitus, dysphonia, convulsions, and herpes zoster recurrence was reported.	[10]

**Table 3:** Case studies with reported SEs of COVID-19 vaccinations.

Vaccine	Cases	Patient	Date/Duration	Complication/Side Effects	References
mRNA-based COVID-19 immunization	Case I (Pfizer BioNTech)	73-year-old female	16 days after the first dose	Acute zoster ophthalmicus (HZO) in right V1 dermatome	[55]
	Case II (Pfizer BioNTech)	69-year-old female	10 days after the first dose	HZO in left V1 dermatome	
	Case II (Moderna)	72-year-old female	13 days after the first dose	An eruption in the left V1 dermatome	
Inactivated COVID-19 immunization	7 cases (Sinopharm COVID-19)	30–55 year seven patients (3 males)	Within 15 days of first dose	Ocular adverse effects: episcleritis, anterior scleritis, acute macular neuroretinopathy, acute middle maculopathy, subretinal fluid	[56]
mRNA-based COVID-19 immunization	One case (Pfizer BioNTech)	80-year-old female on hemodialysis for two and half years	4 days after the first dose	Takotsubo cardiomyopathy with LV outflow tract obstruction	[57]
mRNA-based COVID-19 immunization	One case (Pfizer BioNTech)	29-year- old male	6 days after the first dose	Multiple cranial neuropathy	[58]
Inactivated COVID-19 immunization	One case (Sinopharm COVID-19)	18-year-old female	5 days after second dose	Anterior uveitis associated with juvenile idiopathic arthritis (JIA)	[59]
viral vector-based COVID-19 immunization	Two cases (AstraZeneca vaccine)	-	-	Transverse myelitis is a neurological disorder; unlikely to be related to the vaccine as the patient already had multiple sclerosis	[10]
mRNA-based COVID-19 immunization	One case (Pfizer- BioNTech)	29-year-old female	16 days after the second dose	Autoimmune side effect: antineutrophil cytoplasmic autoantibody (ANCA)-associated vasculitis of acute kidney injury	[60]
mRNA-based COVID-19 immunization	Case I (Pfizer- BioNTech COVID-19 vaccine)	53-year-old male with no previous history	8 days after the second dose	Severe immune thrombocytopenia (ITP); platelet count of 2 × 10^9^/L	[61]
	Case II (Pfizer- BioNTech COVID-19 vaccine)	67-year-old male previous chronic ITP patient but no history of recent flares of ITP	2 days after the first dose	Severe ITP; platelet count of 2 × 10^9^/L	
Viral vector-based COVID-19 immunization	Case III (Janssen COVID-19 vaccine)	59-year-old female with a history of chronic ITP	2 days after the first dose	Severe ITP; platelet count of 64 × 10^9^/L	
mRNA-based COVID-19 immunization	One case (Pfizer- BioNTech COVID-19 vaccine)	82-year-old female	2 weeks after the first dose	Guillain-Barré syndrome (GBS) with generalized body aches, paresthesia, and difficulty walking	[62]
mRNA-based COVID-19 immunization	Case I (Pfizer- BioNTech COVID-19 vaccine)	47-year-old female	15 days after first dose	Lympho-proliferative disease: left infra-clavicular non-painful lump along with fatigue, myalgia, and mild pyrexia to 38 °C	[63]
	Case II (Pfizer- BioNTech COVID-19 vaccine)	46-year-old female	5 days after first dose	Left supraclavicular and axillary painful multiple enlarged lymph nodes, along with headaches, chills.	
	Case III (Pfizer- BioNTech COVID-19 vaccine)	42-year-old female	18 days after the first dose	Left axillary lymph nodes up to 2 cm in diameter	
mRNA-based COVID-19 immunization	Case I (Moderna COVID-19 vaccine)	64 -year-old female with a history of shellfish allergy	Within 10 min of the first dose	Hypersensitivity reaction with generalized pruritus, urticaria, and self-reported tachycardia	[64]
	Case II (Moderna COVID-19 vaccine)	39-year-old female with history of allergic rhinitis	Within 15 min of the first dose	Hypersensitivity reaction with chest and neck urticarial and mild facial angioedema	
Inactivated COVID-19 immunization	One case (Sinopharm COVID-19)	18-year-old female with a history of antinuclear antibody positive oligoarticular juvenile idiopathic arthritis (JIA)	5 days after the second dose	Bilateral anterior uveitis with reduced visual acuity in both eyes	[59]
Viral vector-based COVID-19 immunization	One case (Janssen COVID-19 vaccine)	74-year-old male	3 days after the dose	Severe cutaneous adverse reaction with panhypopituitarism secondary to craniopharyngioma resection, vision loss of the left eye, neurogenic bladder, and obstructive sleep apnea	[65]
Viral vector-based COVID-19 immunization	One case (Janssen COVID-19 vaccine)	41-year-old morbidly obese gentleman	Within four weeks of dose	Guillain-Barré syndrome (GBS)	[66]
mRNA-based COVID-19 immunization	One case (Moderna COVID-19 vaccine)	72-year-old female	1 day after receiving the first dose	Idiopathic thrombocytopenic purpura with a rash, spontaneous oral bleeding, headache and easy bruising or abnormal bleeding.	[67]
mRNA-based COVID-19 immunization	Case I (Moderna COVID-19 vaccine)	74-year-old female	8 days after the first dose	COVID arm: pruritic, erythematous plaque with mild scaling on her left upper arm, rash spread to 15 cm in diameter over 10 days	[68]
	Case II (Moderna COVID-19 vaccine)	62-year-old female	8 days after the first dose	COVID arm: Pruritic erythematous rash on her left deltoid, began as a maculopapular eruption over the injection site	
	Case III (Moderna COVID-19 vaccine)	54-year-old female	7 days after the first dose	COVID arm: Erythematous, non-scaly patch on her left upper arm	
	Case IV (Moderna COVID-19 vaccine)	72-year-old female	10 days after the first dose	COVID arm: Erythematous patch on left deltoid surrounding the injection site, pruritic, warm to the touch, and measured 14 cm in diameter	
mRNA-based COVID-19 immunization	Case I (Moderna COVID-19 vaccine)	77-year-old male with a history of Psoriasis and Crohn’s Disease	2 days after the first dose	Shingles (herpes zoster) with severely painful, unilateral dermatomal herpetiform eruptions	[69]
	Case II (Pfizer- BioNTech COVID-19 vaccine)	65-year-old male	After second dose	Shingles (herpes zoster) with painful, erythematous, clustered skin eruptions and pruritus	
mRNA-based COVID-19 immunization	Case I (Pfizer- BioNTech COVID-19 vaccine)	58-year-old male	1 day after first dose	Varicella-zoster virus reactivation: herpetiform umbilicated vesicle with fever and cervical lymphadenopathy	[70]
	Case II (Pfizer- BioNTech COVID-19 vaccine)	47-year-old female	5 days after the first dose	Varicella-zoster virus reactivation: herpetiform umbilicated vesicle with fever and dysesthesia	
	Case III (Pfizer- BioNTech COVID-19 vaccine)	39-year-old male	3 days after the first dose	Varicella-zoster virus reactivation: painful Herpetiform umbilicated vesicles	
	Case IV (Pfizer- BioNTech COVID-19 vaccine)	56-year-old female	2 days after the second dose	Varicella-zoster virus reactivation: herpetiform umbilicated vesicle with fever and dysesthesia	
	Case V (Pfizer- BioNTech COVID-19 vaccine)	41-year-old female	16 days after the second dose	Varicella-zoster virus reactivation: herpetiform umbilicated vesicle with fever and dysesthesia	
mRNA-based COVID-19 immunization	Case I (Moderna COVID-19 vaccine)	52-year-old male	2 weeks after the dose	Anti-neutrophil cytoplasmic antibody (ANCA) glomerulonephritis	[71]
	Case II (Moderna COVID-19 vaccine)	39-year-old male	Immediately after second dose	Acute kidney injury (AKI) with nephritic syndrome, de novo vasculitis	
	Case III (Moderna COVID-19 vaccine)	81-year-old male	Mild after the first dose, worsened after the second dose	AKI, proteinuria, de novo vasculitis	
mRNA-based COVID-19 immunization	Case I (Moderna COVID-19 vaccine)	86-year-old female	7 days after the dose	Encephalopathy associated with non-convulsive status epilepticusat, poor neurological function, acute confusion, Visual hallucinations, Left frontal headache	[72]
	Case II (Moderna COVID-19 vaccine)	73-year-old male	7 days after the first dose	Encephalopathy associated with non-convulsive status epilepticusat, staring episodes, restlessness, cognitive deficits.	
mRNA-based COVID-19 immunization	Case I (Pfizer- BioNTech COVID-19 vaccine)	64-year-old woman with chronic idiopathic thrombocytopenic purpura (ITP)	2 days after the first dose	Acute immune thrombocytopenia with oral bleeding and generalized petechiae.	[73]
	Case II (Pfizer- BioNTech COVID-19 vaccine)	61-year-old woman with scleroderma	After the second dose	Acute immune thrombocytopenia with petechiae on both legs after.	
	Case III (Moderna COVID-19 vaccine)	73-year-old woman	11 days after the first dose	Acute immune thrombocytopenia with generalized petechiae.	
mRNA-based COVID-19 immunization	Case I (Moderna COVID-19 vaccine)	35-year-old Caucasian woman, stable history of clinically isolated demyelinating syndrome (CIS),	Twenty-one days after the second dose	CNS demyelination: New neurologic symptoms with ataxia/dysmetria in the right upper extremity, and mild gait ataxia, with an Expanded Disability Status Scale (EDSS) score of 2.5.	[74]
	Case II (Moderna COVID-19 vaccine)	26-year-old white Hispanic woman no significant past medical history	Fourteen days after the second dose	CNS demyelination: New visual symptoms involving the right eye, mild blurring, progressed to worsening blurriness and pain with eye movement OD.	
	Case III (Pfizer- BioNTech COVID-19 vaccine)	24-year-old Vietnamese woman	One day after the second dose	CNS demyelination: Presented with new onset left eye vision changes; visual symptoms in the right eye with blurred vision and pain on eye movement with monocular decreased visual acuity.	
	Case IV (Pfizer- BioNTech COVID-19 vaccine)	64-year-old Caucasian man with no history of neurologic diseases,	Eighteen days after the first dose	CNS demyelination: Pain and paresthesia in his upper abdomen progressed to right lower extremity numbness, weakness, pain and numbness in the bilateral lower extremities, saddle anesthesia, sphincter dysfunction, and balance/gait difficulty.	
	Case V (Pfizer- BioNTech COVID-19 vaccine)	33-year-old Caucasian man with no significant past medical history	One day after the second dose	CNS demyelination: Unilateral painless blurring of vision with visual acuity of 20/50 OS and multiple T2 hyperintense white matter lesions on brain MRI.	
	Case VI (Moderna COVID-19 vaccine)	44-year-old Caucasian woman with a medical history of MS at age 20 when	Six days after the second dose	CNS demyelination: Transient low-grade fever with new neurological symptoms including numbness that ascended from her feet to the middle of her waist without any bowel or bladder incontinence. EDSS score of 1.5 with mild right deltoid and iliopsoas weakness.	
	Case VII (Pfizer- BioNTech COVID-19 vaccine)	48-year-old Caucasian woman with a history of the Clinically isolated demyelinating syndrome (CIS)	15 days after the first dose	CNS demyelination: developed with a painful sensation behind her right eye, worsening with eye movement; Brain MRI showed three new T2 hyperintense white matter lesions compared to prior imaging 2 years earlier.	
Inactivated COVID-19 immunization	One case (CoronaVac, Sinovac)	A 76-year-old female	1 day after vaccination	Acute asthma exacerbation with multiple infiltrations in both lungs and ground-glass shadows in both lung fields.	[75]

## Data Availability

Not applicable.
